# Prenatal genetic diagnosis of tetrasomy 18p from maternal trisomy 18p: a case report

**DOI:** 10.1186/s13039-022-00602-4

**Published:** 2022-06-27

**Authors:** Can Peng, SiYuan LinPeng, Xiufen Bu, XuanYu Jiang, LanPing Hu, Jun He, ShiHao Zhou

**Affiliations:** Department of Genetics and Eugenics, Changsha Hospital for Maternal & Child Health Care Affiliated to Hunan Normal University, Changsha, 410007 Hunan China

**Keywords:** Trisomy 18p, Tetrasomy 18p, Isochromosome 18p, Prenatal diagnosis

## Abstract

**Background:**

Tetrasomy 18p syndrome is a rare chromosomal disorder that is caused by the presence of isochromosome 18p. Most tetrasomy 18p cases are de novo cases and maternal origin of trisomy 18p is a rare condition. At present, only four cases of maternal origin have been reported in worldwide.This is the fifth case of tetrasomy 18p originating from maternal trisomy 18p. The mother of the fetus studied had no apparent disease phenotype.

**Case presentation:**

The current case report is to describe a fetus with confirmed 18p tetrasomy as detected by karyotyping and Single Nucleotide Polymorphism array (SNP array) analysis. However, the fetus showed normal phenotypic features that were observed using ultrasound scans. The mother and maternal grandfather were phenotypically normal and healthy; however, they were diagnosed with trisomy 18p, which was confirmed by conventional karyotyping and SNP array.

**Conclusions:**

We report a case of 18p tetrasomy in a fetus whose mother and grandfather had 18p trisomy. The mother and grandfather were phenotypically normal. Our case report findings provide an important reference for the genetic counseling of trisomy 18p in the future.

## Background

Tetrasomy 18p syndrome (OMIM#614,290) is a very rare chromosomal disorder with a prevalence ranging from 1/140,000–1/180,000 [1], and it affects both sexes equally. Tetrasomy 18p syndrome is associated with developmental delays, cognitive impairment, microcephaly, hypertonia, strabismus, scoliosis/kyphosis, and anatomical variants detected on brain MRI scans [1]. Tetrasomy 18p is caused by the presence of isochromosome 18p, which consists of two copies of the short arm of chromosome 18. The isochromosomes are supernumerary chromosomes that are composed of two copies of the same arm of a chromosome. Isochromosome 18p is one of the most commonly observed isochromosomes. Most isochromosome 18p cases are de novo, while a few cases are of maternal origin. At present, only four cases of tetrasomy 18p of maternal origin have been reported [2–5]. A few past reports have discussed that the mechanism of isochromosome 18p development may be related to maternal meiosis II nondisjunction and centromeric misdivision, or U type strand exchange [6–9]. In this case study, we report a family where the mother and grandfather had an 18p trisomic disorder and appeared phenotypically normal, and the fetus inherited an 18p tetrasomic disorder.

## Case presentation

**Fig. 1:**
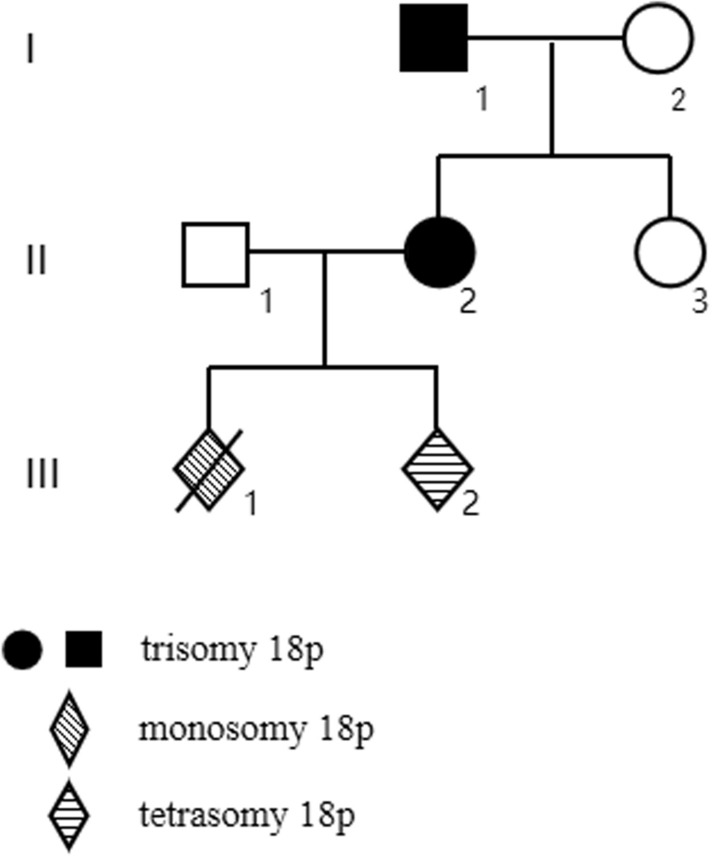
The pedigree. I1: grandfather, I2: grandmother, II2: mother, II3: aunt

**Fig. 2:**
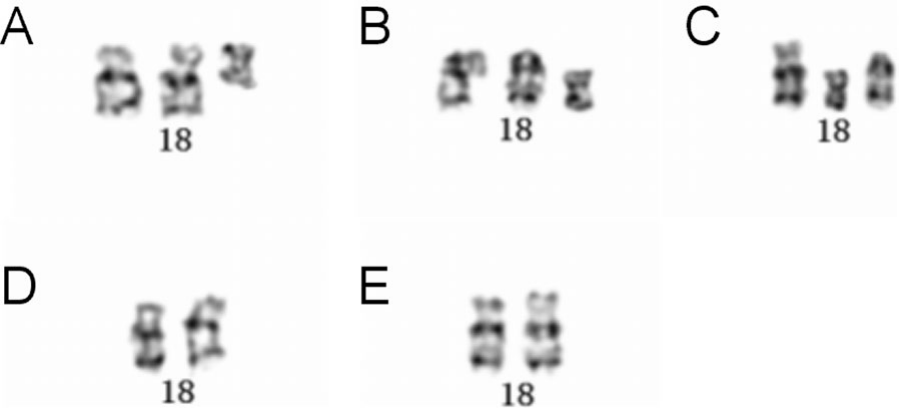
Partial karyograms showing only chromosome 18 and its derivatives for all studied individuals. (**A**) 47,XN, + i(18)(p10) in individual III-2; (**B**) 47,XX,del(18)(p11), + i(18)(p10) in individual II-2; (**C**) 47,XY,del(18)(p11), + i(18)(p10) in individual I-1; (**D**) 46,XX in individual I-2; (**E**) 46,XX in individual II-3

**Fig. 3:**
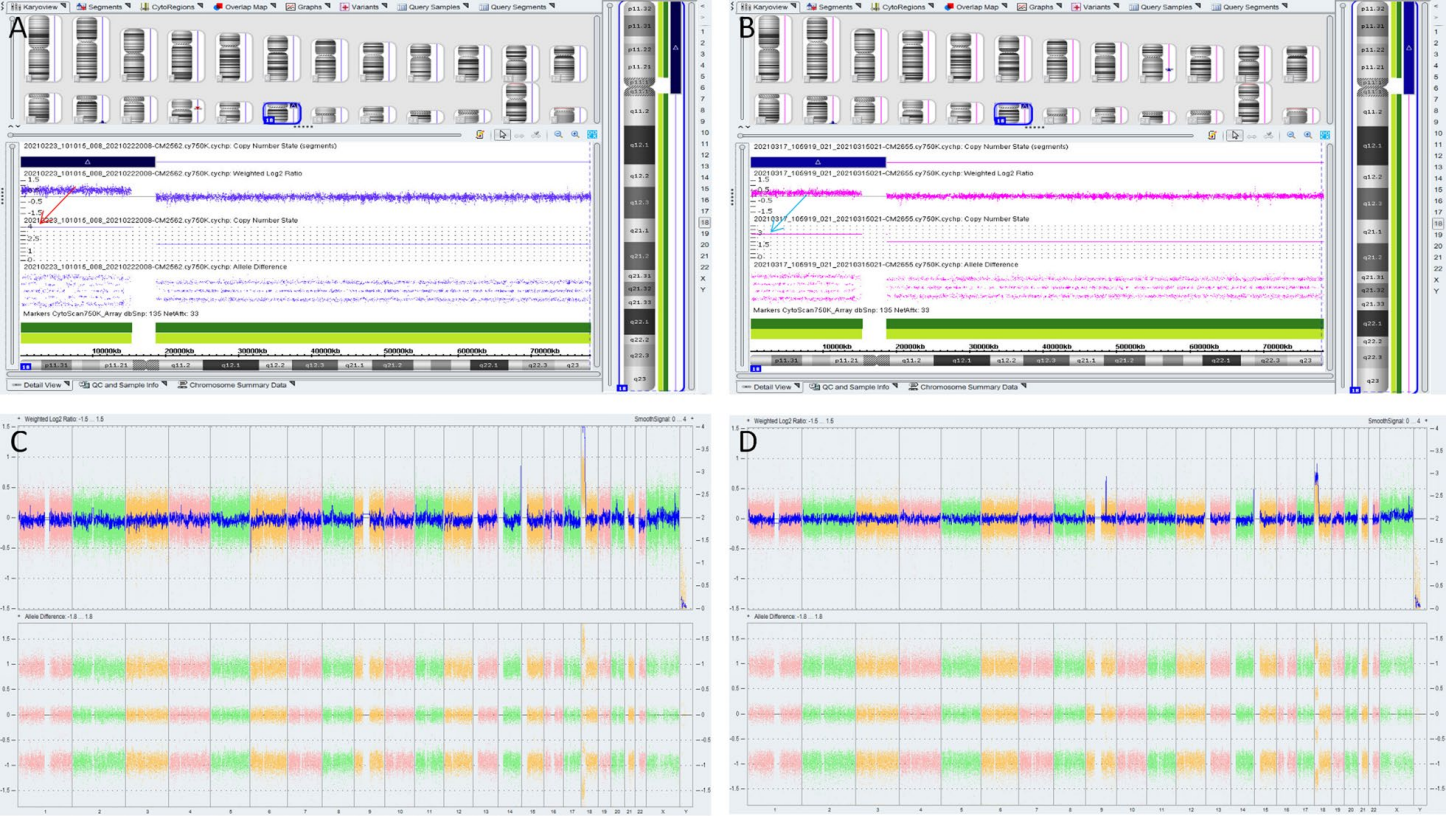
The results of SNP array analysis. (**A-C**) The fetal amniotic fluid sample SNP array revealed an tetraploid of 18.4 Mb of the 18p11.32p11.1 region (red arrow); (**B-D**) The mother’s peripheral blood sample SNP array revealed an 18.4 Mb duplication of the 18p11.32p11.1 region (Pathogenic CNV) (blue arrow), and a 1.29 Mb duplication of the 9q22.2 [variant of unknown significance (VUS)]

A 23-year-old woman was referred to the Department of Medical Genetics at Changsha Hospital for Maternal and Child Health Care, for genetic counseling. The woman,a gravida 2, had a history of pregnancy complications occurring between 17 and 18 weeks of gestation, one of which included one that resulted in induced abortion. In 2019, after 5 months of gestation, she underwent induced labor because "fetal nuchal cystic hygroma" was detected in the fetus; these processes were performed at a different hospital(The Maternal and Child Health Hospital of Hunan Province).The hospital performed copy number variation sequencing (CNV-seq) of the fetal tissue, which revealed a 14.82 Mb deletion of the 18p11.32p11.21 region, and a 1.32 Mb duplication of 9q22.2. Subsequently, amniocentesis was performed, and G-banding karyotype analysis, with a band resolution of 320 bands, of the cultured amniocytes revealed an unusual type of tetrasomy 18p: 47,XN, + i(18)(p10) [10] (Fig. 2A). Microarray analysis of the uncultured amniocytes, using Affymetrix CytoScan 750 K SNP array, revealed arr[GRCh38] 18p11.32q11.1(136,227_20,954,823) × 4 (Pathogenic copy number variants [CNV]) (Fig. 3A). Afterward, karyotyping and SNP array analysis were performed on specimens from pregnant women from the same family, and only karyotyping was performed on the rest of the family. The mother of the fetus’s karyotype (Fig. 1II_2_) was 47,XX,del(18)(p11), + i(18)(p10) (Fig. 2B), and the result of the SNP array analysis was arr[GRCh38] 18p11.32q11.1(136,227_20,966,972) × 3 (Pathogenic CNV) and arr[GRCh38] 9q22.2(89,761,883_90,992,862) × 3 (variant of unknown significance (VUS) CNV) (Fig. 3B). The woman was 158 cm tall and weighed 44 kg. Upon examination, her head circumference, face, lungs, heart, abdomen, spine, and limbs were normal. She graduated from junior middle school, but her competence in mathematics was low. Thereafter, she worked as a kindergarten teacher. The maternal grandfather’s karyotype (Fig. 1I1) was 47,XY,del(18)(p11), + i(18)(p10) (Fig. 2C). He worked as a welder, and also had a low competence in arithmetic. The maternal grandmother (Fig. 1I2) and aunt (Fig. 1II_3_) had normal karyotypes; they were phenotypically normal and healthy (Fig. 2D and E). Prenatal ultrasounds did not detect any abnormalities in the fetus during the last pregnancy. The woman decided to terminate the pregnancy after genetic counseling. Unfortunately, no autopsy was performed on the fetus.

## Discussion and conclusion

Tetrasomy 18p syndrome is a rare type of chromosomal syndrome with phenotypic heterogeneity. The majority of tetrasomy 18p patients experience overall global developmental delay, seizures (21.5%), and feeding difficulties (56.1%). Additionally, they have dysmorphic features, abnormal brain MRI (63%), central hypotonia (49.6%), acquired microcephaly (47.7%), strabismus (45%), cryptorchidism (17.4%), scoliosis/kyphosis (37%), recurrent otitis media (34.6%), and congenital heart disease (23.7%) [11, 12]. Furthermore, tetrasomy 18p patients have lower bone mineral density than that of normal healthy individuals [13], recurring eye and ocular adnexa, and distinctive facial features associated with tetrasomy 18p [14]. Due to the lack of specific morphologic features that characterize tetrasomy 18p syndrome, a prenatal ultrasound diagnosis of tetrasomy 18p syndrome is difficult due to the absence of obvious malformations. Therefore, an ultrasound combined with prenatal genetic testing is an effective method for the diagnosis of tetrasomy 18p syndrome. In this report, prenatal diagnosis conducted due to the history of pregnancy complications. There was no obvious abnormality in early ultrasound scans possibly due to the early gestational age, or the limitations of ultrasound detection. The woman decided to terminate the pregnancy after receiving genetic counseling. Unfortunately, no autopsy was performed on the fetus; thus, we cannot add partial phenotype to this report.

Tetrasomy 18p is caused by the presence of isochromosome 18p, which consists of two copies of the short arm of chromosome 18. Most isochromosome 18p cases are de novo, and a few cases are of maternal origin. Taylor et al. [2] studied a mother with trisomy 18p syndrome and without significant phenotypic abnormality, who had two children: one with monosomy 18p syndrome and another with tetrasomy 18p syndrome.Moreover, Takeda et al. [3] described a phenotypically normal mother, with a karyotype described as 47,XX,del(18)(p11), + i(18)(p10), who had two daughters with tetrasomy 18p syndrome. Furthermore, Abeliovichet et al. [4] reported a female diagnosed as a mosaic for i(18p) with a karyotype 46,XX, + i(18p) [2]/46,XX[58]. The female had a normal cognitive function and completed secondary school education. Her daughter inherited the abnormal chromosome 18 and was diagnosed with tetrasomy 18p syndrome. Boyle et al. [5] reported tetrasomy 18p syndrome in two maternal half-sisters; their mother had a normal phenotype, and her results from karyotyping and fluorescence in situ hybridization analysis of peripheral blood and skin fibroblasts were normal. The authors speculated that it was likely caused by maternal gonadal mosaicism. The four cases of tetrasomy 18p syndrome were of maternal origin, and all the mothers had no apparent disease phenotype. In this case report, the woman had no obvious abnormal phenotype and had a low competence in mathematics, which was consistent with previously reported findings. In addition, this report analyzed the karyotypes of the other family members and confirmed that the grandfather transmitted two abnormal copies of chromosome 18 to the offspring: one copy was missing the short arm of chromosome 18, and the other was isochromosome 18p. The grandfather has no apparent abnormal phenotype but was not competent in arithmetic skills. The grandfather had seven siblings; none of them had a history of abnormal pregnancies. It was speculated that the two abnormal chromosomes were de novo in origin. The mechanism of formation of isochromosome 18p is through misdivision of the centromere or a U type strand exchange during mitotic or meiotic division [8].

Trisomy 18p is a rare chromosomal aberration. The phenotypic spectrum of patients with trisomy 18p is wide, and cognitive function ranges from normal intelligence to mild intellectual disability. According to the pathogenesis of chromosomal aberrations, isolated trisomy 18p can be divided into four types: unbalanced translocation, small supernumerary marker chromosomes (sSMCs), 18p deletion, and isochromosome 18p duplication. Thirty-six cases of isolated trisomy 18p have been published in the literature [15–18], and the clinical phenotypes of most cases were intellectual disability, developmental delay, distinct facial features, epilepsy syndromes, and short stature. Seven of these cases were phenotypically normal. In our case report, the phenotypes of the mother and grandfather of the fetus were normal: this is because the critical region for trisomy 18 phenotype, which has been established at 18q, is preserved in most trisomy 18 cases. The role of environmental factors on phenotype requires further study.

The sSMCs simultaneously represent structural chromosomal abnormalities as well as numerical ones. 2% sSMC carriers showed (almost) normal outcomes, most likely due to mosaicism [19]. According to Dr. Liehr’s database (http://ssmc-tl.com/sSMC.html) [accessed 5/2022]), 387 cases of isochromosome 18p have been reported. The cases in this study enrich the database.

In our report, we described a phenotypically normal family with an 18p trisomic mother and grandfather and an 18p tetrasomic fetus. Our case report adds descriptive information on the cohort of patients with trisomy 18 and could provide a reference for genetic counseling in the future.

## Data Availability

All data generated and analyzed during this study are included in this published article.

## Abbreviations

SNP array: Single nucleotide polymorphism array
CNV-seq: Copy number variation sequencing
CNV: Copy number variants
sSMCs: Small supernumerary marker chromosomes
